# *Staphylococcus aureus* Host Spectrum Correlates with Methicillin Resistance in a Multi-Species Ecosystem

**DOI:** 10.3390/microorganisms11020393

**Published:** 2023-02-03

**Authors:** Barthelémy Ngoubangoye, David Fouchet, Larson Amédée Boundenga, Cécile Cassan, Céline Arnathau, Helene Meugnier, Thierry-Audrey Tsoumbou, Serge Ely Dibakou, Désiré Otsaghe Ekore, Yasmine Okomo Nguema, Nancy Diamella Moukodoum, Arsene Mabicka, Tristan Ferry, Jean Phillipe Rasigade, Franck Prugnolle, Anne-Laure Bañuls, François Renaud, Dominique Pontier

**Affiliations:** 1 Centre Interdisciplinaire de Recherches Médicales de Franceville (CIRMF), Franceville BP 769, Gabon; 2 CNRS, Laboratoire de Biométrie et Biologie Evolutive UMR5558, Université de Lyon 1, 69622 Villeurbanne, France; 3 LabEx ECOFECT, Eco-Evolutionary Dynamics of Infectious Diseases, Université de Lyon 1, 69622 Villeurbanne, France; 4 Laboratoire MIVEGEC, UMR-CNRS 5290-IRD 224, IRD Montpellier, 34394 Montpellier, France; 5 Centre National de Référence des Staphylocoques, Institut des Agent infectieux, Hôpital de la Croix Rousse, Hospices Civils de Lyon, 69004 Lyon, France; 6 Laboratoire de Bactériologie, Observatoire Rhône-Alpes des Mycobactéries, Institut des Agents Infectieux, Hospices Civil, 69004 Lyon, France; 7 Centre International de Recherche en Infectiologie, INSERM U1111, CNRS UMR5308, Université de Lyon 1, ENS de Lyon, Hospices Civil Lyon, 69004 Lyon, France

**Keywords:** natural ecosystem, humans, non-human primates, bats, micromammals, strain biodiversity, generalist and specialist strains, antibiotic resistance

## Abstract

Although antibiotic resistance is a major issue for both human and animal health, very few studies have investigated the role of the bacterial host spectrum in its dissemination within natural ecosystems. Here, we assessed the prevalence of methicillin resistance among *Staphylococcus aureus* (MRSA) isolates from humans, non-human primates (NHPs), micromammals and bats in a primatology center located in southeast Gabon, and evaluated the plausibility of four main predictions regarding the acquisition of antibiotic resistance in this ecosystem. MRSA strain prevalence was much higher in exposed species (i.e., humans and NHPs which receive antibiotic treatment) than in unexposed species (micromammals and bats), and in NHP species living in enclosures than those in captivity—supporting the assumption that antibiotic pressure is a risk factor in the acquisition of MRSA that is reinforced by the irregularity of drug treatment. In the two unexposed groups of species, resistance prevalence was high in the generalist strains that infect humans or NHPs, supporting the hypothesis that MRSA strains diffuse to wild species through interspecific transmission of a generalist strain. Strikingly, the generalist strains that were not found in humans showed a higher proportion of MRSA strains than specialist strains, suggesting that generalist strains present a greater potential for the acquisition of antibiotic resistance than specialist strains. The host spectrum is thus a major component of the issue of antibiotic resistance in ecosystems where humans apply strong antibiotic pressure.

## 1. Introduction

Antibiotic resistance is currently a major problem in public health at the global level and results primarily from the selection pressure exerted on bacteria by the overuse of antibiotics [1,2,3] although natural resistance unrelated to human activity is increasingly being reported [4,5,6]. While the use of antibiotics in hospital is well known to be a major issue in drug resistance, the use of antibiotics in animals, such as those maintained in captivity and semi-captivity (e.g., in zoos and parks), for therapeutic use, or food production (poultry, pigs, and cows) is now considered to be an important factor in the emergence and spread of drug resistance [7,8]. Although much is known regarding how resistant bacteria spread in hospitals, i.e., within single host species communities [9,10], the scale of the problem, and factors that influence the emergence, dissemination and persistence of antibiotic-resistant bacteria in natural ecosystems where humans are in close contact with domestic animals or wildlife, i.e., in multi-host species communities [11,12,13,14] require investigation. As bacterial pathogens can cross species barriers, it is critical to understand the connections between human and animal (domestic and wild) microbiota to manage this One Health challenge [11,15,16,17].

In natural ecosystems, an important feature of the problem of antimicrobial resistance is the host spectrum of the microorganisms [18,19]. Indeed, intuitively, it is reasonable to assume that, when a species A is exposed to an antibiotic pressure that leads to favor resistance in its bacterial populations, while a species B is not, then the level of resistance observed in species B should be higher when resistant bacteria infecting A are able to cross the A/B species barrier, i.e., are generalist. The strength of selection imposed by the host ecological community and the cost of being generalist (capable of infecting more than one host species) or resistant shape the evolution and coexistence of different strategies. Bacteria evolving in a structured host community might experience a variable antimicrobial environment across host species. For example, in a two species system with a treated and an untreated species in close contact and equal proportions, specialist strains (infecting a single-host species) will suffer strong and low antibiotic pressure, respectively, while generalist strains will suffer intermediate pressure. In other words, generalist strains suffer an antibiotic pressure that is the average of the pressures exerted on all the hosts in its spectrum, and thus are expected to exhibit intermediate levels of antibiotic resistance when they infect species with different levels of antibiotic exposure. By contrast, specialist strains, which suffer only the pressure linked to the exposition of their (unique) host species, may more easily exhibit more extreme (high or low) levels of antibiotic resistance. So, the microorganism’s host spectrum, which has been largely overlooked in the study of antimicrobial resistance, may thus be an important trait to consider for characterizing antimicrobial resistance in natural ecosystems.

In Africa, excessive and inappropriate antibiotic consumption has led to antimicrobial resistance through a postulated mechanism of antibiotic selection [20,21]. In veterinary medicine, the observation is the same due to systematic use of antibiotics as preventive and curative treatment [22]. Among human pathogens of critical priority [21,23], *Staphylococcus aureus* is one of the most common in Africa [24]. *S. aureus* is both a commensal and pathogen of humans and various mammalian species [25,26]. It is part of the normal skin and nasal microbiota, with approximately 30% of humans permanently colonized [27]. Symptomatic infection can occur if there is a breach in the mucosal barrier or skin, which can cause a wide variety of infections ranging from mild to life-threatening disease, such as bacteremia, infective endocarditis or thrombophlebitis [28,29]. Mechanisms of the occasional spread of *S. aureus* from lesions are not fully understood, although several risk factors have been identified for the development of sepsis (e.g., age, additional comorbidities [30]. The spread of methicillin-resistant *S. aureus* (MRSA; see Supplementary Table S1 for a full list of non-canonical abbreviations used in the manuscript) has followed the use of different types of antibiotics over the years and now poses a serious problem in anti-microbial chemotherapy worldwide [31]. In addition to their increasing prevalence and incidence, MRSA infections are associated with rising morbidity and mortality, in comparison to methicillin-susceptible *S. aureus* (MSSA) infections [10,32,33]. Furthermore, the bacterium has the ability to switch host [29,34,35,36] even after long periods of isolation in a single host species [37].

In this study we examine the relationships between host spectrum and antibiotic resistance in *S. aureus*. To do this, we analyzed the population structure of methicillin-resistant *S. aureus* (MRSA) in host populations (humans, non-human primates, micromammals, and bats) in the Primatology Center (Centre de Primatologie, CDP) at the Interdisciplinary Center for Medical Research in Franceville (CIRMF), Gabon (Central Africa). The CDP offers an ecosystem where species submitted to different antibiotic pressures coexist: humans are more exposed to antibiotics than NHPs and micromammals (rodents and shrews), whereas bats are unexposed. Within the NHP species present, an important contrast is also found in the regularity of treatment, species living in aviaries are under full control of veterinarians while treatments can be erratic or interrupted for species living in enclosures because of difficulties in recapturing animals. The ecosystem is also diverse regarding bacterial characteristics, and, as we will see below, show that both specialist and generalist strains coexist.

The objective of this study is to take advantage of this natural but human-driven ecosystem to test predictions (see Material and Methods section) about the frequency of MRSA strains. To this end, we isolated *S. aureus* strains from humans, NHPs, bats and micromammals, typed their *spa* gene [38], and identified MRSA by amplifying the *mecA* gene [39].

## 2. Material and Methods

### 2.1. Predictions

Our first prediction (P1) is that humans, then NHPs, the two species that benefit from medical care, show a higher prevalence of MRSA than bats and micromammals, which are not directly exposed to antibiotics. The second prediction (P2) concerns repercussions of the incorrect use of antimicrobials upon the selection of resistant strains. Irregularities in treatment administration could lead NHP species living in enclosures to exhibit a higher prevalence of MRSA than NHP species living in captivity (aviaries). Bearing in mind that many other factors are not controlled, any observed differences between the two groups could also be due to other mechanisms. The third prediction (P3) examines the link between the host spectrum and the prevalence of MRSA. Even though we cannot exclude the hypothesis of SCCmec acquisition by non-resistant specialist strains, considering the stability of MRSA [40], in species not exposed to antibiotic pressure (bats and micromammals), only the generalist strains infecting exposed species (humans and/or NHPs) should show significant levels of MRSA. We also expect that, in NHPs and humans, generalist strains that also infect micromammals or bats will show a lower level of antibiotic resistance than specific strains. Finally, micromammals are an interesting group because their small dispersal distance (compared to bats) should constrain the geographical spread of the bacterium. The prevalence of drug resistance may decrease with distance from a site of antimicrobial pressure [1] here, buildings housing NHPs or humans. However, the role of the host spectrum in this phenomenon has not been studied. If drug resistance imposes a substantial cost on the transmissibility of the bacterium [41], we predict [P4] that being a generalist strain also impacts the efficacy of its transmission in rodents [42]. To test this, we test whether three quantities decrease with the distance from NHP installations: (i) the proportion of MRSA strains in infected individuals; (ii) the proportion of MRSA strains in individuals infected by generalist strains infecting exposed species; and (iii) the proportion of generalist strains infecting exposed species in infected individuals. The corresponding hypothesis for bats, which fly over long distances, was not investigated.

### 2.2. Host Community Studied and Sampling

**Figure 1:**
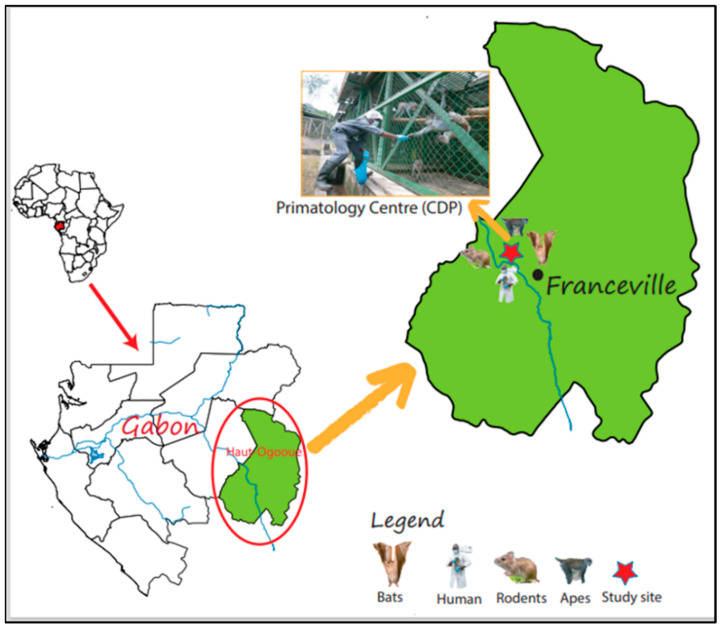
Location of study site in Gabon. The Primate Center (CDP: 1°37′59″ N/13°34′59″ E) is in the southeast of Gabon.

The research was conducted during 2013 and 2014 in the Primatology Center (CDP) of the Interdisciplinaire Center for Medical Research in Franceville, Gabon (CIRMF). The CDP is located in the city of Franceville in the Haut-Ogooué province, southeast Gabon (1°37′59″ N/13°34′59″ E; Figure 1). The closest habitations are less than 500 m from the CDP. At the time, the CDP hosted more than 350 NHPs belonging to eight species including chimpanzees (*Pan troglodytes troglodytes*), gorillas (*Gorilla gorilla gorilla*), mandrills (*Mandrillus sphinx*) and five other monkey species (*Cercocebus torquatus*, *Chlorocebus aethiops*, *Macaca* sp., *Allochracebus solatus*, *Cercopithcus cephus*). A team of 22 people provided care, feeding and monitoring the animals. NHPs are housed in two distinct environments. The first group of species (*M. sphinx*, *C. solatus* and *C. cephus*) live in forested enclosure of six hectares whereas the second group (*Pan t. troglodytes*, *Gorilla g. gorilla* and other monkeys) live in large aviaries. Moreover, during the night, frugivorous bats, rodents and shrews feed on leftover food—in particular bananas—in the feeding areas (4 tonnes of bananas are received each week at the CDP to feed NHPs).

### 2.3. Ethics Statement

All protocols (human and animals) concerning this study were approved by the Gabonese National Ethics Committee (Authorization N°PROT/0020/2013I/SG/CNE). All work (captures, euthanasia and animal handling) was performed following the guidelines of the American Society of Mammalogists (http://www.mammalsociety.org/committees/animal-care-and-use, accessed on 14 March 2014). Captured animals were removed carefully as soon as possible to minimize injury, drowning, strangulation, or stress.

As recommended by the Gabonese National Ethics and Research Committee for micromammals and bats, safe euthanasia was practiced through the use of inhalant anesthetic (halothane). Finally, human samples were collected by the CIRMF medical team based on the workers of primatology center voluntary participation. All healthy workers participating in the study provided their own informed written consent.

### 2.4. Isolation and Identification of S. aureus

Nasal swabs were collected from the nose of humans, non-human primates, bats and rodents. For micromammals and bats, we used swabs adapted for small animals. All samples were streaked on SAID and blood agar plates according to the manufacturer’s instructions (bioMérieux, Marcy l’Etoile, France). Isolates were identified on the basis of colony morphology, Gram staining, catalase tests, and coagulation tests (SAID, Marcy l’Etoile, France) and verification was carried out by PCR for the presence of *nuc* (a species-specific marker) as described previously [43].

### 2.5. Spa Typing Analysis

DNA sequencing of short sequence repeats (SSR) of the polymorphic X region of the protein A gene (*spa*) was used to type all *S. aureus* isolates [44,45]. PCR amplification of the SSR area of the *spa* gene was accomplished as described previously [45,46]. DNA sequencing of the PCR-amplified products (150–500 bp) was performed by Eurofins MWG (Germany). The standard chromatogram files of the forward and reverse sequences obtained from each sample were edited and assembled and *spa* types were determined using Ridom StaphType software v.2.1.1 (Ridom GmbH, Würzburg, Germany; [47]). All sequences were verified using the online BLAST database (http://blast.ncbi.nlm.nih.gov/Blast.cgi, accessed on 17 November 2016). Identified *spa* types with similar repeat profiles (i.e., sharing identical SSR units arranged in an identical order) were clustered into *spa* clonal complexes (*spa*-CCs) using the BURP-algorithm (Staph Type v2.1.1 software, Ridom GmbH, Münster, Germany [47]). *Spa* types shorter than 5 repeats were excluded (because their information content is limited and no reliable evolutionary history can be inferred) [48,49]; a cluster composed of 2 or more related *spa* types was regarded as a clonal complexe; a *spa* type that was not grouped into a clonal complexe was considered a singleton. Specific sequence types (STs) were assigned according to *spa* types reported on the Ridom Spa Server database (http://spa.ridom.de/spatypes.shtml, accessed on 8 December 2016).

### 2.6. Amplification of the mecA Gene

DNA was extracted from positive S. aureus samples (amplification of nuc gene by PCR) using the DNeasy Blood and Tissue Kit (Qiagen, Courtaboeuf, France SAS) according to the manufacturer’s instructions. The bacterial colony suspension was used as a DNA template to identify Methicillin-resistant S. aureus by PCR-amplifying the mecA gene, which encodes the modified penicillin binding protein PBP2a [50].

### 2.7. Statistical Analyses

To test the P1, differences of proportions in *S. aureus* carriage and MRSA between types of hosts (NHPs, humans, micromammals and bats) were assessed using a chi-square test. If significant, the global chi-square test was followed by a post-hoc analysis comparing species pairwise using a Bonferroni correction where the *p*-value (*p*) is multiplied by the number of tests.

For the other three predictions, we used a logistic regression model in *S. aureus* positive individuals (non-infected individuals were removed from the data set for the analyzes associated with predictions P2, P3 and P4) with MRSA carriage as a response variable (in all analyzes but P3b, see below). Cofactors considered were sex (s), age as a categorical variable (a: young, juvenile and adult for NHPs; juvenile and adult for micromammals and bats; in humans age was replaced by seniority in the work-place, sw), species (spe, only for NHPs, rodents and bats), distance from NHP installations (d, only for micromammals), housing (h, i.e., enclosures or aviaries, only for NHPs) and the degree of generalism (g, a simplified indicator of the host spectrum).

We classified worker seniority into three groups: people working at the CDP for less than 5 years, 5–10 years, and more than 10 years. In micromammals, distance from NHP installations was defined as the minimum distance between the trap where the individual was captured and the nearest NHP installation (aviary or enclosure). Finally, the degree of generalism was considered as follows. In humans, due to the small sample size, we considered only two modalities: a strain was considered specialist when found only in humans, and generalist otherwise. In NHPs, specialist strains were those infecting only NHPs. Generalist strains were divided into two groups: those also infecting humans (whether or not they infected bats or micromammals; group GH) and those infecting other species but not humans (group GnH). In bats and micromammals, two groups were also separated: strains only infecting untreated species (micromammals and bats; group GnE) and those also infecting humans or NHPs, i.e., at least one of the two species that are directly exposed to antibiotics (group GE).

For P2, on NHP MRSA data, we started by choosing the set of correcting cofactors. From the model s*a+g, we selected models using the Akaike Information Criterion (AIC) as recommended by Burnham and Anderson [51], and retained the submodel with the lowest AIC score. This allowed us to retain a limited combination of correcting cofactors that have a good predictive power. Then, species (spe) was added to the model as a random effect. Housing was then added to the model and its effect was tested using a likelihood ratio test (LRT).

For P3, in humans and NHPs, MRSA data were considered separately. We first performed an AIC procedure on submodels of a full model (s*sw in humans, s*a+spe in NHPs and bats, and s*a+spe+d in micromammals) to select a combination of correction cofactors in each data set. From the selected model, variable g was introduced into the model and parameters associated with this variable were estimated together and tested equal to zero, the modality “specialist” of variable g being taken as the intercept—so the estimated parameters quantify the difference in the probability (quantified by the odd ratios) of the acquisition of resistance between specialist and generalist strains (according to their host spectrum).

For P4, on micromammal MRSA data, we first performed an AIC procedure on the submodels of s*a+spe to select a combination of correction cofactors. Then, distance (d) was added to the model and its associated coefficient was estimated using a maximum likelihood procedure. The significance of the estimated coefficient was not tested because, from a biological point of view, it is not relevant. Indeed, even if we assume no cost of resistance, the flow of entry of resistant strains with the micromammal population is asymmetric since it is highly likely that most of these strains enter the micromammal population from buildings housing NHPs or humans.

In this first analysis associated with P4 (analysis P4a), the estimate of the coefficient associated with d evaluates how the frequency of resistant strains changes with the distance from human installations. Host spectrum was neglected here intentionally, to see what we would have concluded had we neglected this variable.

Because most resistant strains in our data set are also GE strains (see results for P3), we then considered whether the change of resistance frequency with distance from human installations was most attributable to changes in the frequency of GE strains or in the frequency of resistant strains among GE strains. To disentangle the two phenomena, we performed the same analysis using (i) the full data set with the variable GE (that equals 1 if the strain is GE and otherwise 0, i.e., if the strain is GnE or S) as a response variable (analysis P4b). The estimate of the coefficient associated with d in this model informs us as to how the frequency of GE strains changes with d and (ii) a restricted data set considering only GE strains with MRSA carriage as a response variable (analysis P4c). The estimate of the coefficient associated with d in this latter model informs us as to how the frequency of resistant strains changes with d in GE strains.

For analyzes P4b and P4c, we performed the same preliminary AIC procedure (on the submodels of s*a+spe) to select a combination of correction cofactors, then added distance (d).

Statistical analysis was performed with R software v3.1.0. All statistical tests were performed with a 5% type 1 risk. For each group of species, coefficient estimates of the model presenting the lowest AIC of all potential combination of cofactors considered is presented in Supplementary Tables S3 and S4.

## 3. Results

### 3.1. Prevalence of S. aureus in the Host Community

**Figure 2:**
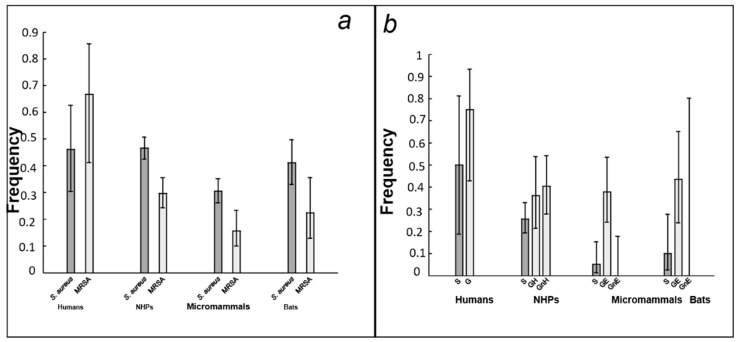
Difference in *S. aureus* and MRSA carriage between four different groups of species. (**a**) Prevalence of *S. aureus* (dark gray) and relative prevalence (among individuals infected by *S. aureus*) of MRSA (light gray) carriage in four groups of hosts; (**b**) Relative prevalence of MRSA carriage in groups of hosts according to host spectrum. Strains are separated according to whether they are specialists (S, dark gray) or generalists (G, light gray). In NHPs, generalist strains were separated into two groups: those that also infect humans (strains GH) and those that do not (strains GnH). In micromammals and bats, generalist strains were separated into two groups: those that also infect exposed hosts (humans and/or NHPs, strains GE) and those that do not (strains GnE).

1180 different individuals were sampled (39 humans, 580 NHPs, 141 bats and 420 micromammals). All the 1180 nasal swabs collected were examined, and 474 were identified correctly as containing *S. aureus* (Supplementary Table S2). Globally, the proportion of positive individuals on *nuc*-PCR was 46% in humans, 46.6% in NHPs, 41% in bats and 30.5% in micromammals (Supplementary Table S2; Figure 2a). No significant differences between humans and NHPs (*p* = 1), between humans and bats (*p* = 0.705) or between humans and micromammals (*p* = 0.067) were detected (Table 1). No significant differences were observed between NHPs and bats (*p* = 0.287). However, NHPs were significantly more infected than micromammals (*p* = 0.02 × 10^−10^) (Table 1).

### 3.2. Spa Typing

The number of identified repeats ranged between 1 (t693, t779, t1781) and 11 (t148, t12895, t15967). Eight *spa* types were excluded due to their low number of repeats (<5). BURP analysis resulted in two *spa*-CCs that have been described previously (CC2546 with 2 *spa* types grouping 20 isolates, CC15941 with 2 *spa* types grouping 48 isolates in generalist and specialist strains, respectively). The other isolates were identified as singletons (7 *spa* types among generalist strains and 16 *spa* types among specialist strains, representing 130 and 103 isolates, respectively), or as groups with no founder (2 for generalist strains and 10 for specialist strains, representing 32 and 70 isolates, respectively) (Table 2 and Table 3). 

### 3.3. S. aureus Population Structure and Distribution among Hosts

In total, 474 sequences were examined of which 14 were unusable because of their bad sequence quality. Forty-nine different *spa* types were identified from the remaining 460 isolates. The distribution of *S. aureus* strains among host species and their characteristics are given in Table 2 and Table 3. Globally, 198 isolates representing 13 *spa* types were shared between different hosts (generalist strains, Table 2) and 262 isolates representing 36 *spa* types were isolated from one species only (specialist strains, Table 3). Four specialist *spa* types in humans corresponded to 6 isolates, 14 in NHPs corresponded to 168 isolates (13 from *P. t. troglodytes*, 34 from *Macaca* sp., 118 from *M. sphinx* and 1 from *G. g. gorilla*, *C. solatus* and *C. aethiops*), 10 in bats corresponded to 30 isolates (2 from *E. helvum*, 22 from *E. franqueti*, 5 from *H. monstrosus* and 1 from *R. aegyptiacus*) and 8 in micromammals corresponded to 58 isolates (8 from L. striatus, 9 from *L. nudicaudus*, 22 from *M. musculus*, 8 from *Praomys* sp., 10 from *R. rattus* and 1 from shrew). Thirteen *spa* types were shared between two or three host species: NHPs and humans (t537, t189), NHPs and bats (t2546, t15942, t5725), NHPs and micromammals (t056, t084), micromammals and bats (t15969, t1781), NHPs, humans and bats (t355), NHPs, micromammals and bats (t5017), humans, micromammals and bats (t939) (Table 2).

### 3.4. Testing the Four Predictions for MRSA Carriage

Our first prediction (P1) was that the prevalence of MRSA is higher in species benefiting from medical care (humans and NHP) than in bats and micromammals. Consistently, we found significant differences in MRSA carriage between the four groups of species (*p* = 1.8 × 10^−5^, see Table 1). MRSA prevalence was significantly higher in humans (67%, 95% confidence interval: [41%; 86%]) than in the three other groups of species. Prevalence was then the highest in NHPs (30%, 95% CI [24%; 36%]), then in bats (22%, 95% CI [13%; 36%]), and lowest in micromammals (16%, 95% CI [10%; 23%]) (Figure 2a).

Our second prediction (P2) was that NHP species living in enclosures in which antibiotic protocols are harder to follow exhibit a higher prevalence of MRSA carriage than those living in captivity (aviaries). We kept the degree of generalism (g) as well as the multiplicative effects of age (a) and sex (s) as correction factors (Table S3) to test the difference in MRSA carriage between species living in enclosures and aviaries (Table 4). The difference between the two classes of housing was significant (*p* = 0.01) and the estimated coefficient supported the prediction that individuals living in enclosures show the highest rate of MRSA carriage (OR = 3.19 [1.51 to 6.75]) (Table 4).

The base model was determined using an AIC procedure (Supplementary Table S3). The modality ‘aviaries was taken as the reference class of the ‘housing’ variable, so the odd-ratio gives the increased risk of MRSA carriage in individuals living in enclosures compared to individuals living in aviaries.

Our third prediction (P3) was that the prevalence of MRSA carriage is linked with host spectrum. Most specifically it is higher when the spectrum includes exposed species and lower when it includes non-exposed ones. Consistently, MRSA carriage was significantly linked to the host spectrum in NHPs, bats and micromammals (Table 5, Supplementary Table S4 and Figure 2b). In humans the link was not significant. In NHPs, both generalist strains (GH: OR = 2.5 [1.02; 6.17] and GnH: OR = 2.33 [1.09; 5]) were more resistant than specialist strains. In bats, MRSA carriage was much higher in generalist strains infecting host species exposed to antibiotics (GE: OR = 6.89 [1.65 to 28.78]) than in specialist strains. A similar pattern was observed in micromammals (GE: OR = 10.05 [2.67; 37.82]).

**Figure 3:**
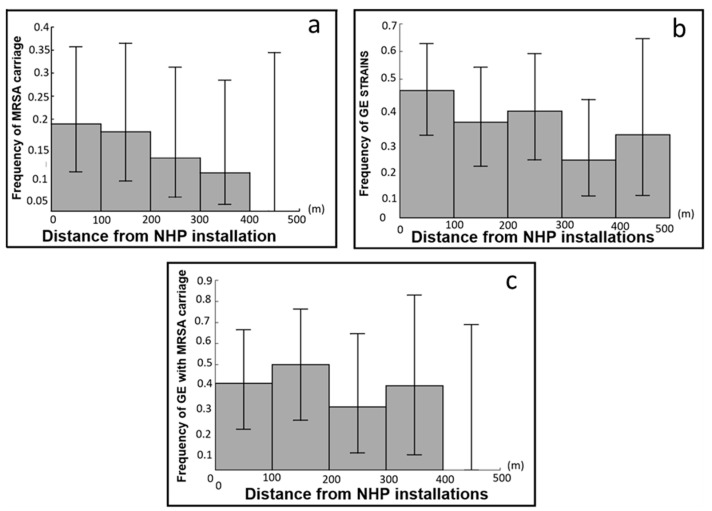
Effect of the distance (in meters) from NHP installations (*x*-axis) in micromammals on (*y*-axis) (**a**) the proportion of individuals carrying MRSA strains among individuals infected by *S. aureus*; (**b**) the proportion of GE strains among individuals infected by *S. aureus* and (**c**) the proportion of individuals carrying MRSA strains among individuals infected by GE strains.

Finally, our last prediction (P4) was that the frequency of MRSA carriage in micromammals decreases with the distance to human installations, a phenomenon that could be also partly due to the decay of generalist strains. We estimated the intensity of the link between MRSA carriage and distance (d) from a NHP installation for micromammals (analysis P4a, host spectrum neglected in this analysis) with no correction factors (Supplementary Table S3). After adding d to the base model, the associated coefficient revealed a negative link between d and the prevalence of MRSA (for each 100 m increase in d, odds ratio = 0.59 [0.38; 0.92], Table 6). This decrease is substantial, but individuals infected by resistant strains were still found more than 300 m from NHP installations (Figure 3a)

Since most MRSA strains in micromammals (17/20) were generalist strains also infecting exposed species (GE strains), the decay in the proportion of MRSA strains in infected individuals with distance to PHN installations (Figure 3a) could also be due to a decrease in the frequency of GE strains in infected individuals with distance from NHP installations. This was indeed the case (Figure 3b). To quantify the importance of the decay in the proportion of GE strains in infected individuals, we performed analysis P4b. We kept sex as a correction factor (Supplementary Table S3) and found that for each 100 m increase in distance from a NHP installation, there is an odds ratio for GE strains in infected individuals of 0.74 ([0.54; 1.02], Table 6). In GE strains, the decay of the frequency of MRSA strains with d was quite similar (Figure 3c and analysis P4c: odds ratio for each 100 m increase in d = 0.72 [0.44; 1.19], no correction factor, see Supplementary Table S3).

## 4. Discussion

Few studies of primatology have focused on NHPs in sanctuaries and in general studies have been limited to interactions between humans and NHPs [34,52]. The primary originality of our study was to analyze *S. aureus* population structure and antibiotic resistance in a broader ecosystem context composed of phylogenetically distant hosts: humans, NHPs, bats and micromammals living in proximity in a primatology center in Gabon. The second originality was to include host spectrum as a potential critical factor for understanding the distribution of MRSA strains among hosts dwelling in this ecosystem.

### 4.1. MRSA Strain Prevalence

The prevalence of *S. aureus* in our human population is quite high (46%) but consistent with other data for Africa. The prevalence of *S. aureus* in humans is variable both within (e.g., from 2 to 52.6% in Kenya) [53] and between countries in Africa, and can be as high as 63.1% in Eritrea [54]. Differences in the characteristics of specific study populations, the type of specimens collected, types of healthcare facility and methodological differences are well known factors that explain variability in *S. aureus* prevalence [54]. Our study also found a high rate (66.7%) of MRSA colonization among humans. Similar values are found in other countries in Africa (up to 82%, Falagas et al. 2013), but generally in caregiver and patient populations [55,56]. The high prevalence of MRSA in our study can be explained in two ways. (i) Firstly, by the lack of regulation of antibiotic consumption in Gabon, like in other African countries [55,56,57], where antibiotics can be purchased without medical prescription. (ii) Secondly, our sample is a small group of workers in the CIRMF who spend most of their working time together, and, because they usually work with non-human primates are generally afraid of being infected by a zoonotic disease. They commonly self-medicate by consuming antibiotics at the slightest fatigue (B.N., personal observation).

The most prevalent MRSA isolates in previous studies in Gabon were t084 (ST15) and t355 (ST152) isolates. t084 isolates made up 35% of all isolates responsible for *S. aureus* carriage in inhabitants of Lambaréné and surrounding villages (500 km from Franceville) [58]. In another study, performed in Foumagou (244 km from Franceville), ST15 strains colonized 30% of mothers and 25% of their children and ST152 strains colonized 25% of mothers and 22% of their children [59]. In NHPs, the diversity of *S. aureus* carriage has never been explored in Gabon, although ST15 and ST152 strains have been previously detected in a limited number of animals (domestic animals and *Cephalophus* sp. [34,60]. In our study, these isolates were not as highly prevalent in humans as they are in animals, but we found that ST15 strains were generalist in NHPs and micromammals, and ST152 strains were generalists in humans, NHPs and bats.

Focusing on MRSA clones, hospital-associated clones, such as ST239 and ST241, have been previously described in nine African countries [61,62,63], but not in our study. Community-acquired MRSA clones usually harbored PVL genes (one of the important virulence factors of *S. aureus*) more frequently than hospital-acquired MRSA clones [61,64]. The MRSA ‘African clone’ ST88 (t186), which is highly prevalent in West, Central, and East Africa as well as Madagascar [61], does not harbor PVL genes. This clone has been significantly associated with *S. aureus* infections in Gabon (5 isolates were found in pygmy populations [65] whereas the prevalence of this clone is very low (only one patient in the study of Ateba Ngoa et al. [58]. We found this clone in only 2 humans, and not in animals.

The most prevalent generalist *S. aureus* strains were ST188 (t189), t939, t5017, t15942, and t084 (Table 2), the last four of unknown ST. The strain t15942 was found in 17 NHPs and 5 bats, but not in humans. Importantly, 15 of the t15942 strains collected (68%) were MRSA, suggesting circulation of a MRSA clone in animals. Regarding specialist strains, the most prevalent strain with the highest proportion of MRSA (47%) was t15943 (Table 3), of unknown ST. This strain was found in 34 NHPs. Further studies are needed to determine if this strain is a new clone as well as its epidemic potential, not only in each animal species but also between animals and humans.

### 4.2. Antibacterial Drug Pressure and the Importance of the Host Spectrum

MRSA strains were found in all groups of host species. In line with prediction P1, MRSA prevalence was significantly different between host groups of species, resistant strains being more prevalent in humans. However, surprisingly, we found relatively high prevalence of MRSA in bats and micromammals, two taxa that are not exposed to antibiotic treatment. The host spectrum could well be the explanation for these results.

In the two unexposed (bats and micromammals) groups of species, resistance carriage was very low in specialist strains (Table 3) and absent in the generalist strains that were not found in exposed species (i.e., humans and NHPs: GnE) (t1781 and t 15969, Table 2 and Figure 2b). Conversely, resistance prevalence was high in the generalist strains that were able to infect humans or NHPs (GE), at rates that were comparable to what is observed in NHPs (Table 5 and Figure 2b). This result shows the important role of antibiotic pressure in resistance acquisition. If human-driven selection for antibiotic resistance through environmental contamination occurs (some specialist strains of micromammals and bats carried resistance genes, but resistance could also have been acquired through horizontal gene transfers from resistant generalist strains), its impact is much lower.

If we combine these two unexposed species, of the 33 MRSA infected individuals, 5 (15%) were carrying a strain classified as specialists. The most straightforward explanation for this result is a misclassification of the host spectrum of these strains. Here we considered a strain to be specialist when it was not found in any other species, but, of course, not finding it does not mean it does not exist. So, these strains could be misclassified generalists.

In micromammals, we assumed that contact points with human installations represented hotspots of MRSA strain acquisition. For characters that imply a gain of function for a parasite (such as antibiotic resistance or generalism), it is quite common (and reasonable) to assume that the acquisition of the function is associated with a reduction of fitness (e.g., of transmissibility) in conditions where this function is not useful [66]. As a result, we expected the frequency of MRSA strains to decrease with distance from human installations. Such a phenomenon has been observed for the anthropogenic exposure of *E. coli* to ticarcillin (β-lactams) [1].

Such decay was also observed in our study (although not statistically tested), but, more interestingly, it was relatively slow. MRSA strains were still found with a non-negligible frequency in micromammals > 300 m away from human installations. One possible explanation could be that contact points with human installations are not be the only places where MRSA transmission occur. For example, wastewater might transport antibiotics and the bacteria over hundreds of meters from the place of use [13,67] or contaminated bats could have contacts with micromammals inside the forest. This latter explanation seems unlikely in our case because we did not observe any MRSA strains infecting both rodents and bats.

In contrast, it is also possible that the distance at which we observed MRSA infected micromammals simply translates fitness costs. In this case, the relatively slow decay of the prevalence of resistance strains with distance could suggest that the fitness reduction of resistant strains is not particularly large with the result that they continue to spread between individuals before finally fading out.

In addition, because almost all resistant strains are generalist—and yet have no clear advantage far from human installations because only one host is present—the decay in MRSA prevalence with distance from human installations could also arise from the decay of GE strains with distance. This was indeed the case, and we observed that the decay of MRSA strain prevalence with distance from human installations appeared to be attributable to both a decay in the prevalence of generalist strains and to that of resistant strains among generalist strains in roughly equivalent proportions.

Our work has important limitations. In particular, the determination of the host spectrum is uncertain. We already discussed the fact that finding a strain in only one host species is not a definitive argument to declare it a specialist. In addition, *spa* typing is not sufficiently discriminative to follow the dissemination of strains when they spread between humans and animals. However, we believe that introducing our (imperfect) estimation of host strain spectrums in our analysis brought interesting insights that raise important questions. For example, how strongly are resistant strains counter-selected once antibiotic pressure is released? Since the transmission of resistant strains could be due to the inter-species transmission of generalist strains to some extent, what is the relative cost of generalism compared to that of resistance once both antibiotic pressure and inter-specific transmission opportunities are released?

The results we have discussed up to this point are consistent with our predictions. This means that the picture thus far is consistent with the intuitive ecological view we have about the spread antibiotic resistance between exposed and unexposed species. However, NHPs show a more intriguing scenario. In NHPs, MRSA carriage was significantly higher in species living in enclosures than in aviaries, supporting prediction P2. The interpretation of this result is not straightforward because the living conditions of individuals differ between the two environments, so many factors may explain this phenomenon. Contrasts in the regularity of treatment administration between the two groups may explain these differences (NHPs in enclosures are less accessible to regular veterinary care following an infection than those in aviaries), which raises the question of the importance of applying the correct antibiotic regimen to limit the spread of resistance strains [68].

Regarding host spectrum, we had little a priori knowledge of the impact of generalism on the spread of MRSA in NHPs. Spreading through human hosts could be an additional source of resistance acquisition. Thus, MRSA carriage could be higher in generalist strains. But resistance in generalist strains could also be counter-selected through the spreading of *S. aureus* in bats and micromammals. Generalism may thus also reduce MRSA carriage.

To understand the link between host spectrum and MRSA, we separated generalist strains found in NHPs according to whether they were also found in humans or not. Generalist strains infecting humans were significantly more resistant than specialist strains, which was consistent with what we expected. However, strikingly, generalist strains that were not found in humans were also more resistant than specialist strains (Table 5 and Figure 2b). This result is highly counter-intuitive because, while spreading to bats or micromammals, the release of antibiotic pressure should have decreased the level of resistance. At best, generalist strains that were not found in humans should have had the same level of resistance as specialist strains. This result may suggest that generalist strains, beyond the pressures imposed by their ecological trajectory (in terms of host species), are more prone to acquire resistance to methicillin. One possible hypothesis is that they are more adaptable than specialist strains. This hypothesis makes sense with regard to the ecological constraints imposed on these strains. Generalist strains, when they change host, need to adapt to a new environment. Thus, these strains may be more ‘adaptable’ to antibiotic pressures, for example through faster acquisition of resistance or decreased associated fitness cost. Interestingly, bacteria using a generalist strategy are structurally different to specialist strains [69]. These structural differences may be important in the acquisition of resistance. For the moment this hypothesis remains speculative, but, if true, it could have important epidemiological implications. If generalist strains are more prone to acquire antibiotic resistance, antibiotic pressure could be an important factor for bacterial transmission between species by favoring strains with a broader host spectrum. Thus, for example, the treatment of livestock could, indirectly, leads to an increased risk of bacterial transmission to humans [70].

## 5. Conclusions

This study demonstrates the interest of considering host spectrum as a fundamental trait to better understand the problem of drug resistance in multi-host species ecosystems. In terms of conservation, the results show that sanctuaries such as the Primatology Center can promote the transmission of *S. aureus* strains between host species and that drug resistance can be spread to non-treated species. This can have consequences for the effectiveness of antibiotics in human and animal health. In sanctuaries or conservation programs, the application of specific procedures is required to limit the spread of antibiotic resistance. The guidelines presented by the IUCN (International Union for Conservation of Nature) [71]) and GRASP (Great Apes Survival Partnership) should move in this direction. Regarding human health, many ecosystems involving treated and untreated species arise from the husbandry of animals by humans (zoos, public parks, stock farms, etc.) everywhere in the world. Research is needed to understand the role of animal biodiversity and the spectra of hosts in the spread and persistence of antibiotic resistance in such anthropogenic ecosystems.

## Figures and Tables

**Table 1:** Comparison of *S. aureus* and MRSA carriage between host type species by chi-square test or Fisher’s exact test.

	Host	N° Isolates			*p*			
		Positive	Negative	N	Human	NHPs	Bats	Microm.
*S. aureus* carriage	Humans	18	21	39	−	1	0.705	0.067
	NHPs	270	310	580	−	−	0.287	0.02 × 10^−10^
	Bats	58	83	141	−	−	−	0.15
	Microm.	128	292	420	−	−	−	−
	Global comparison		Chi-square = 26.89		df = 3			*p*-value = 6.2 × 10^−6^
MRSA carriage	Humans	12	6	18	−	0.016	0.008	0.008
	NHPs	80	190	270	−	−	0.340	0.02
	Bats	13	45	58	−	−	−	0.35
	Microm.	20	108	128	−	−	−	−
	Global comparison		Chi-square = 24.61		df = 3			*p*-value = 1.8 × 10^−5^

Significance of the global chi-square test was followed by a post-hoc analysis comparing species pairwise using a Bonferroni correction where the *p*-value is multiplied by the number of tests. The abbreviation ‘Microm’. represents micromammals and MRSA represents methicillin resistance among *S. aureus* (MRSA).

**Table 2:** Generalist *spa* types. *Spa* type shared between different hosts and classified according to antibiotic-exposed (Humans and NHPs) and -unexposed species (micromammals and bats). Each *S. aureus spa* type is indicated with associated ST (Specific Sequence type), *spa* cluster analysis (CC = clonal complex), the *spa* profile (SSR = Short Sequence Repeats), the number of strains per species infected, with the number of MRSA strains amongst them in brackets. In bold, the new strains described in this study and reported to the Ridom SpaServer website (http://spa.ridom.de/spatypes.shtml, accessed on 18 January 2017). *Spa* types with less than 5 repeats were not analyzed. The abbreviation ‘Microm.’ stands for micromammals.

*Spa* CC	*Spa* Type	Associated ST	SSR Profile	Hosts				Total
				Exposed Species		Unexposed Species		
				NHPs	Human	Microm.	Bats	
2546	t2546	None	04-12-17-20-17-12-17-17	1(**0**)			4(1)	5(1)
	t056	ST101	04-20-12-17-20-17-12-17-17	6(**3**)		9(5)		15(8)
Singleton	t537	None	07-23-12-21-12-20-17-12-12	9(1)	1(0)			10(1)
	**t15942**	None	**08-13-17-20-17-25-17-13**	17(12)			5(3)	22(15)
	**t15969**	None	**388-12-17-12-17-12-17-174-16-13**			11(0)	1(0)	12(0)
	t5017	None	08-34-02-43-34-43-43-16-02-17-83	13(4)		10(6)	3(1)	26(11)
	t189	ST188	07-23-12-21-17-34	20(8)	3(3)			23(11)
	t939	None	04-16-34-12-34-12		3(3)	15(6)	4(2)	22(11)
	t1458	None	121-21-17-17-23-24		1(0)	10(0)	4(1)	15(1)
No founder	t084	ST15-ST18	07-23-12-34-34-12-12-23-02-12-23	18(3)		1(0)		19(3)
	t355	ST152/377/1633	07-56-12-17-16-16-33-31-57-12	7(4)	4(3)		2(1)	13(8)
Not analysed	t1781	None	26-16-16			12(0)	1(0)	13(0)
	t5725	ST72	121	2(1)			1(1)	3(2)
**Total**	**13**			93(36)	12(9)	68(17)	25(10)	**198(72)**

**Table 3:** Specialist *spa* type. *Spa* type isolated from one species only and classified according to antibiotic-exposed (Humans and NHPs) and unexposed species (micromammals and bats). Each *S. aureus spa* type is indicated with associated ST (ST = Specific Sequence type), *spa* cluster analysis (CC = clonal complex), the *spa* profile (SSR = Short Sequence Repeats), the number of strains per infected species, with the number of MRSA strains amongst them in brackets. In bold, the new strains described in this study and reported to the Ridom SpaServer website (http://spa.ridom.de/spatypes.shtml, accessed on 18 January 2017). *Spa* types with less than 5 repeats were not analyzed. The abbreviation “microm.’ stands for micromammals.

Exposure to Antibiotics	Characteristic of *spa* Type				Number	Host
**Exposed species**	Spa CC	*Spa* type	Associated ST	SSR profile		
	15941	t13661	None	26-17-17-02-17-12-12-17-16-16	12(0)	NHP
		**t15961**	-	**26-17-17-02-17-12-12-16**	18(3)	NHP
		**t15941**	-	**26-17-17-02-17-12-12-16-16**	17(7)	NHP
	Singletons	t122	-	08-16-02-16-02-25-17-24-24	1(1)	Human
		t148	-	07-23-12-21-12-17-20-17-12-12-17	10(4)	NHP
		t186	ST88	07-12-21-17-13-13-34-34-33-34	3(2)	Human
		t359	-	07-23-12-21-17-34-34-33-34	10(1)	NHP
		t5132	-	08-16-02-16-13-13-17-34-16-13	1(0)	Human
		t7368	-	03-22-31-34-17	13(5)	NHP
		t6980	-	26-16-34-33-13	1(0)	NHP
		**t15940**	-	**121-12-12-34-22**	30(3)	NHP
	No founder	t318	ST30	15-12-16-16-02-16-02-25-17-24	13(2)	NHP
		t1848	-	15-12-17-16-02-16-02-25-17	1(0)	Human
		**t16031**	-	**121-34-22-31-34-17-34-17**	2(1)	NHP
		t6940	-	11-10-17-34-22-25	1(0)	NHP
		t1476	-	11-10-17-34-24-34-22-25	6(1)	NHP
		**t15943**	-	**121-34-34-22-31-34-17-34-17**	34(16)	NHP
	Not analysed	t605	-	07-23	1(0)	NHP
Total Exposed					**174(46)**	
**Unexposed species**	2546	t11341	-	26-30-17-34-17-17-16-12-17-16	1(1)	bat
	Singletons	t094	-	07-23-12-34-34-12-12-23	6(0)	microm
		t3464	-	07-23-02-12-23-02-02-34	9(0)	microm
		t15195	-	388-76-76-12-687-174-16-16-17	1(0)	bat
		**t15962**	-	**121-16-25-16-12-16-13-25-17**	1(0)	bat
		**t15963**	-	**388-12-174-174-16-13-16-13**	1(0)	bat
		**t15964**	-	**391-76-76-12-76-174-16-337-17**	3(0)	bat
		**t15967**	-	**26-349-34-23-96-58-34-82-82-82-24**	4(0)	bat
		**t15973**	-	**26-17-20-17-17-16-16**	9(0)	microm
	No founder	**t15968**	-	**07-56-12-17-16-16-33-31-414-12**	6(0)	bat
		t002	ST5; ST231	26-23-17-34-17-20-17-12-17-16	15(1)	microm
		t2173	-	07-23-17-13-17-20-17-12-17-16	1(0)	microm
		t12895	-	621-23-12-34-34-12-12-23-02-12-23	5(1)	bat
	Not analysed	t586	-	26-16	7(1)	microm
		t693	-	7	4(0)	microm
		t779	-	8	4(1)	bat
		**t15972**	-	**08-21-21-33**	7(1)	microm
		**t15966**	-	**26-12-25-17**	4(0)	bat
Total Unexposed					**88(6)**	
**Total All**		**36**			**262(52)**	

**Table 4:** Likelihood ratio test testing prediction P2 by investigating the link between MRSA carriage and housing in NHPs. The abbreviation ‘spe’ represents species, ‘a’ represents age, ‘s’ represents sex, and ‘g’ represents generalist strains.

Investigated Effect	Base Model	Chi-Square	Df	*p*	Coefficient [95% CI]	Odds Ratio [95% CI]
Housing (enclosure vs. aviaries)	a*s+g + (1|spe)	6.74	1	0.001	1.16 [0.41; 1.91]	3.19 [1.51; 6.75]

**Table 5:** Testing prediction P3 regarding the link between host spectrum (variable g) and the risk of MRSA carriage.

Host	Base Model	Strain	Estimate	Std. Error	z	*p*	Odds Ratio [95% CI]
Humans	H0	Generalist	0.44	1.14	0.38	0.69	1.55 [0.16; 14.44]
NHPs	a+s+spe	GH	0.92	0.46	2.01	0.04	2.5 [1.02; 6.17]
		GnH	0.85	0.39	2.19	0.03	2.33 [1.09; 5]
		Generalist (any)	0.87	0.33	2.63	0.08	2.38 [1.26; 4.53]
Bats	H0	GE	1.93	0.73	2. 61	0.009	6.89 [1.65; 28.78]
		GnE	−∞	NA	NA	0.99	NA
		Generalist (any)	1.79	0.73	2.45	0.01	5.98 [1.43; 25.03]
Microm.	D	GE	2.31	0.68	3.41	0.0006	10.05 [2.67; 37.82]
		GnE	NA	NA	NA	NA	NA
		Generalist (any)	1.717	0.663	2.59	0.0096	5.53 [1.51; 20.43]

The base model was determined using the procedure presented in the Methods section (see Supplementary Table S4 for calculation details). In NHPs, generalist strains were divided into two groups: those also infecting humans (together with bats and/or micromammals or not, group GH) and the others in which humans were not found as infected hosts (group GnH). In bats and micromammals, strains infecting only other unexposed species (micromammals and bats, respectively, group GnE) were separated from those also infecting humans and/or NHPs (exposed species, group GE). In all (groups of) species, specialist strains are used as the reference class, so the odds ratio quantifies how the risk of presenting methicillin resistance is increased in generalist strains compared to specialist strains. ‘Microm.’ represents micromammals, ‘spe’ represents species, ‘a’ represents age, ‘s’ represents sex, and ‘d’ represents distance. NA = Not applicable.

**Table 6:** Testing prediction P4 by estimating the coefficients linking *d* (the distance from human installations) to (i) MRSA carriage in all infected micromammals (analysis P4a); (ii) GE strain carriage in all infected micromammals (analysis P4b) and (iii) MRSA carriage in individuals infected by GE strains (analysis P4c).

Investigated Effect	Group of Infected Hosts	Response Variable	Analysis	Base Model	Coefficient [95% CI]	Odd-Ratio [95% CI]
Distance from human installation	Microm.: all	MRSA carriage	P4a	H0	−0.0052 [−0.0096; −0.008]	0.59 [0.38; 0.92]
	Microm.: all	GE strain carriage	P4b	S	−0.0030 [−0.0061; 0.0002]	0.74 [0.54; 1.02]
	Microm.: GE	MRSA carriage	P4c	H0	−0.0033 [−0.0083; 0.0017]	0.72 [0.44; 1.19]

In each case, the base model was determined with an AIC procedure (Supplementary Table S3). The odds ratios were defined by the exponential of a hundred times the coefficient, giving the increase (more accurately the decrease since the OR is below one) in the risk of MRSA (or GE strain) carriage in micromammals for each 100 m augmentation in the distance from a NHP installation. The abbreviation ‘Microm.’ represents micromammals.

## Data Availability

Not applicable.

## Supplementary Materials

The following supporting information can be downloaded at: https://www.mdpi.com/article/10.3390/microorganisms11020393/s1, Table S1: List of abbreviations; Table S2: Species sampled, *S. aureus* and MRSA strains; Table S3: Generalized linear model (GLM) tested to explain observed variation in MRSA carriage in infected hosts, as well as GE strains carriage (in micromammals for prediction P4); Table S4: General Linear Models (GLM) for prediction P3 in different host groups comparing generalist and specialist MRSA strains.
