# Supplementary material for: Staphylococcus aureus Host Spectrum Correlates with Methicillin Resistance in a Multi-Species Ecosystem
Source: Microorganisms. 2023 Feb 3;11(2):393. doi: 10.3390/microorganisms11020393 (PMC9964919; doi:10.3390/microorganisms11020393)
Supplement: Supplementary file 1 [file microorganisms-11-00393-s001.zip › microorganisms-2072857-supplementary.pdf]

microorganisms

MDPI

Article

Staphylococcus aureus Host Spectrum Correlates with Methicillin Resistance in a Multi-Species Ecosystem

Barthelémy Ngoubangoye [1,2,3,*], David Fouchet [2,3], Larson Amédée Boundenga [1], Cécile Cassan [4], Céline Arnathau [4], Helene Meugnier [5], Thierry-Audrey Tsoumbou [1], Serge Elie Dibakou [1], Désiré Otsaghe Ekore [1], Yasmine Okomo Nguema [1], Nancy Diamella Moukodoum [1], Arsene Mabicka [1], Tristan Ferry [3,6], Jean Phillipe Rasigade [3,7], Franck Prugnolle [1,4], Anne-Laure Bañuls [4], François Renaud [4,†] and Dominique Pontier [2,3,*,†]

[1] Centre Interdisciplinaire de Recherches Médicales de Franceville (CIRMF), Franceville BP 769, Gabon
[2] CNRS, Laboratoire de Biométrie et Biologie Evolutive UMR5558, Université de Lyon 1, F-69622 Villeurbanne, France
[3] LabEx ECOFECT, Eco-evolutionary Dynamics of Infectious Diseases, Université de Lyon, France
[4] Laboratoire MIVEGEC, UMR-CNRS 5290-IRD 224, IRD Montpellier, 34394 Montpellier, France
[5] Centre National de Référence des Staphylocoques, Institut des Agent infectieux, Hôpital de la Croix Rousse, Hospices Civils de Lyon, 69004 Lyon, France
[6] Laboratoire de Bactériologie, Observatoire Rhône-Alpes des Mycobactéries, Institut des Agents Infectieux, Hospices Civil, 69004 Lyon, France
[7] Centre International de Recherche en Infectiologie, INSERM U1111, CNRS UMR5308, Université de Lyon 1, ENS de Lyon, Hospices Civil Lyon, 69004 Lyon, France
* Correspondence: genistha@hotmail.com (B.N.); dominique.pontier@univ-lyon1.fr (D.P.)
† These authors contributed equally to this work.

Supplementary data

Table S1. List of abbreviations.

- Biological terms:
- NHP: Non-human primate
- MRSA: methicillin-resistant Staphylococcus aureus
- CC: Clonal Complex
- SAID: S. aureus identification
- microm: micromammals
- mec A: gene found in bacterial cells which allows them to be resistant methicillin.
- Nuc: amplified gene for S. aureus identification
- SSR: Short Sequence Repeats
- DNA: Deoxyribose Nucleic Acid
- PCR: Polymerase Chain Reaction
- PVL: Panton-Valentine (toxin)
- ST: Sequence Typing
-
- Research structures and Non-governmental organisations:
- CDP: Centre de Primatologie (Primatology Centre)
- CIRMF: Centre International de Recherches Médicales de Franceville (Gabon)
- IRD: Institut de Recherche pour le Développement
- CNE: Centre National d'Ethique (Gabonese National Ethics Committee)
- IUCN: International Union for Conservation of Nature
- GRASP: Great Apes Survival Partnership
-
- Host spectrum:
- S: strains classified as specialist
- G: strains observed in humans and classified as generalist

- GH: strains observed in NHP classified as generalist and that were also observed in humans
- GnH: strains observed in NHP classified as generalist but were not observed in humans
- GE: strains observed in a non exposed species (micromammals or bat), classified as generalist and that were also observed in at least one exposed species (humans or NHP)
- GnE: strains observed in a non exposed species (micromammals or bat), classified as generalist but were not observed in any exposed species (humans or NHP)
-
- Statistical terms:
- AIC: Akaike Information Criterium
- LRT: Likelihood Ratio Test
- OR: Odd Ratio
- factor1 * factor2: interaction between factor1 and factor2
- H0: null hypothesis (model without any explanatory cofactor)
- CI: confidence interval
-
- Cofactors:
- s: sex
- a: age
- spe: species
- d: distance from NHP installations
- h: housing
- g: degree of generalism
- sw: seniority at work

Table S2. Species sampled, *S. aureus* and MSRA strains. *N*= Number of individuals sampled; *Ni*= Number of *S. aureus* isolates among *N*; *Ns* = Number of MRSA isolates among *Ni*. NHPs (Non-Human Primates), "Microm." stands for Micromammals.

Host		Swabs collected		
**Group**	**Species**	***N***	***Ni (%: Ni/N)***	***Ns***
Humans	*H.sapiens sapiens*	39	18 (46.15)	12
NHPs	*Pan t. troglodytes*	83	36	7
	Gorilla g. gorilla	6	5	-
	Mandrillus sphinx	380	180	66
	Cercopithecus torquatus	2	0	-
	Chlorocebus Aethiops	9	1	-
	Macaca sp.	86	43	6
	Cercopithecus solatus	12	4	1
	Cercopithecus cephus	2	1	-
Total NHPs		580	270 (46.55)	80
Bats	*Epomops franqueti*	121	49	11
	Hypsignathus monstrosus	20	9	2
Total Bats		141	58 (41.13)	13
Microm.	*Lemniscomys striatus*	93	18	-
	Lophuromys nudicaudus	52	17	2
	Mastomys natalensis	38	14	2
	Praomys sp	89	24	5
	Rattus norvegicus	98	36	8
	Rattus Rattus	40	16	2

	shrews	10	3	1
Total Micromam.		420	128 (30.48)	20
**Total**		1180	474 (40.17)	125

Table S3. Generalized linear model (GLM) tested to explain observed variation in MRSA carriage in infected hosts, as well as GE strains carriage (in micromammals for prediction P4). The conditions of the models are described in the Methods section. The model with the smallest AIC was retained to test our predictions (in yellow for P2 and P4, in green for P3). Correcting cofactors *a* (age), *s* (sex), *sw* (work seniority), *d* (distance), *g* (generalist or specialist strains of *S. aureus*), *spe* (species). 'NA' means that the inference algorithm failed.

Infected Hosts	Response Variable	GLM	AIC
Humans	MRSA carriage	H0	21.095 (P3)
		s	22.436
		sw	23.095
		s+sw	24.34
		*s*sw*	24.34
Micromammals: all	MRSA carriage	*d+g*	101.50
		s+d+g	103.45
		g	104.09
		d+spe+g	105.31
		s+g	106.08
		esp+g	106.39
		s+d+spe+g	107.13
		a+s+d+g	107.22
		a+g	108.02
		d	108.67 (P3)
		*a*s+d+g*	109.01
		a+s+d+esp+g	109.54
		a+s+g	110.00
		s+d	110.5
		d+esp	111.64
		*a*s+g*	111.93
		*a*s+d+esp+g*	111.98
		a+d	112.65
		H0	112.95 (P4a)
		s+d+spe	113.49
		a+s+d	114.49
		esp	114.79
		s	114.94
		*a*s+d*	116.29
		s+esp	116.78
		a	116.94
		a+s+d+esp	117.08
		a+esp	118.55
		a+s	118.93
		*a*s+d+esp*	119.3
		*a*s*	120.12
		a+s+esp	120.54
		*a*s+esp*	122
Micromammals: all	GE carriage	*s*	165.43 (P4b)
		H0	166.24
		a+s	167.11
		a	167.37
		s+esp	172.44
		esp	172.71

		*a*s*	167.87
		a+esp	174.05
		a+s+esp	174.44
		*a*s+esp*	175.78
Micromammals: infected by GE	MRSA carriage	H0	78.478 (P4c)
		s	79.985
		esp	81.53
		a	82.207
		s+esp	82.522
		*a*s*	83.348
		a+s	83.573
		a+esp	85.156
		a+s+esp	86.255
		*a*s+esp*	NA
Bats	MRSA carriage	H0	63.723(P3)
		s	64.66
		a	67.21
		*a*s*	68.22
		a+s	68.25
		spe	68.68
		s+spe	69.22
		*a*s+spe*	71.83
		a+spe	72.20
		a+s+spe	72.82
NHPs	MRSA carriage	*a+s+spe+g*	264.08
		*a*s+g*	265.39 (P2)
		s+spe+g	265.49
		a+spe+g	265.87
		a+g	267.17
		spe+g	267.57
		s+g	269.52
		g	271.53
		a+s+spe	276.76 (P3)
		a+spe	278.70
		a+s	278.79
		*a*s*	278.85
		a	279.58
		s+spe	280.14
		spe	281.77
		s	283.07
		H0	284.06

Table S4. General Linear Models (GLM) for prediction P3 in different host groups comparing generalist and specialist MRSA strains. GE: generalist strains also infecting humans and/or NHPs, i.e., at least one of the two species that are directly exposed to antibiotics; GnE: generalist strains only infecting untreated species to antibiotics (micromammals and bats); GH: generalist strains infecting humans (whether infecting or not they were also found in bats and/or micromammals); GnH: generalist strains infecting other species but not humans. The abbreviation 'microm.' stands for micromammals.

Hosts	Base Model	Variables	Estimate	Std. Error	z Value	*p*-Value
Humans	H0	*g*	0.44	1.14	0.38	0.69
Microm.	*d*	*d*	−0.0045	0.0024	−1.821	0.068
		g: GE	2.31	0.68	3.41	0.0006 *
		g: GnE	−∞	NA	NA	0.99
Bats	H0	*g*: GE	1.93	0.73	2.61	0.009 *
		g: GnE	NA	NA	NA	NA
NHPs	*a+s+spe*	*age*: adolescent	0.30	0.42	0.70	0.49
		age: juvenile	−1.48	0.80	−1.83	0.07
		sex: male	0.63	0.33	1.92	0.05
		species: *C. cephus*	−15.41	2399.54	−0.006	−0.99
		species: *Pan t. troglodytes*	−1.20	0.52	−2.32	0.02 *
		species: *Gorilla g. gorilla*	−15.90	1085.60	−0.015	0.99
		species: *Macaca* sp.	−1.13	0.51	−2.23	0.03 *
		species: *C. solatus*	−0.06	1.26	−0.05	0.95
		species: *Ch. Aethiops*	−15.41	2399.60	−0.006	0.99
		g: GH	0.92	0.46	2.01	0.04 *
		g: GnH	0.85	0.39	2.19	0.03 *
